# Genome organisation of the *Acinetobacter* lytic phage ZZ1 and comparison with other T4-like *Acinetobacter* phages

**DOI:** 10.1186/1471-2164-15-793

**Published:** 2014-09-14

**Authors:** Jing Jin, Zhen-Jiang Li, Shu-Wei Wang, Shan-Mei Wang, Song-Jian Chen, De-Hai Huang, Gai Zhang, Ya-Hui Li, Xiao-Ting Wang, Jin Wang, Guo-Qiang Zhao

**Affiliations:** Department of Pathogen Biology and Immunology, Henan Medical College, Shuanghu Road #8, Zhengzhou, 451191 P. R. China; Department of Pathogen Biology, Basic Medical College of Zhengzhou University, Kexue Road #100, Zhengzhou, 450001 P. R. China; Clinical Laboratory, Henan Provincial People’s Hospital, Zhengzhou, 450003 P. R. China

**Keywords:** Phage genome annotation, Phage genome organisation, Comparative genomic analyses, T4-like phage

## Abstract

**Background:**

Phage ZZ1, which efficiently infects pathogenic *Acinetobacter baumannii* strains, is the fifth completely sequenced T4-like *Acinetobacter* phage to date. To gain a better understanding of the genetic characteristics of ZZ1, bioinformatics and comparative genomic analyses of the T4 phages were performed.

**Results:**

The 166,687-bp double-stranded DNA genome of ZZ1 has the lowest GC content (34.4%) of the sequenced T4-like *Acinetobacter* phages. A total of 256 protein-coding genes and 8 tRNA genes were predicted. Forty-three percent of the predicted ZZ1 proteins share up to 73% amino acid identity with T4 proteins, and the homologous genes generally retained the same order and transcriptional direction. Beyond the conserved structural and DNA replication modules, T4 and ZZ1 have diverged substantially by the acquisition and deletion of large blocks of unrelated genes, especially in the first halves of their genomes. In addition, ZZ1 and the four other T4-like *Acinetobacter* phage genomes (Acj9, Acj61, 133, and Ac42) share a well-organised and highly conserved core genome, particularly in the regions encoding DNA replication and virion structural proteins. Of the ZZ1 proteins, 70, 64, 61, and 56% share up to 86, 85, 81, and 83% amino acid identity with Acj9, Acj61, 133, and Ac42 proteins, respectively. ZZ1 has a different number and types of tRNAs than the other 4 *Acinetobacter* phages, although some of the ZZ1-encoded tRNAs share high sequence similarity with the tRNAs from these phages. Over half of ZZ1-encoded tRNAs (5 out of 8) are related to optimal codon usage for ZZ1 proteins. However, this correlation was not present in any of the other 4 *Acinetobacter* phages.

**Conclusions:**

The comparative genomic analysis of these phages provided some new insights into the evolution and diversity of *Acinetobacter* phages, which might elucidate the evolutionary origin and host-specific adaptation of these phages.

**Electronic supplementary material:**

The online version of this article (doi:10.1186/1471-2164-15-793) contains supplementary material, which is available to authorized users.

## Background

ZZ1, a novel sequenced lytic phage that can efficiently infect *Acinetobacter baumannii* clinical strains, was identified from fishpond water in Zhengzhou, China [1]. As is typical for T4-like virion morphology, the ZZ1 viral particle contains an isometric head and a contractile tail, which are visible with transmission electron microscopy. The phage was reported to have different antibacterial activity against three *A. baumannii* clinical strains (AB09V, AB0901, and AB0902). Of the host bacteria, AB09V is the most sensitive, and ZZ1 causes large and distinguishable plaque formation on a lawn of AB09V and is highly infectious with a short latent period (9 min) and a large burst size (200 PFU/cell) [1].

To date, the genomes of 12 *Acinetobacter* phages have been completely sequenced and published in the NCBI genome database. Five belong to the *Myoviridae* phage group, including 133, Acj9, Acj61, and Ac42, and four belong to the *Siphoviridae* phage group, including Abp1 [NC_021316.1], Bphi-B1251 [NC_019541.1], AB1 [HM368260.1] and YMC/09/02/B1251_ABA_BP [JX403940.1]. Of the remaining 3 phages, AB3 [NC_021337.1] belongs to the *Podoviridae* phage group; AP205 [NC_002700.2], an ssRNA virus, belongs to the *Levivirus* group; and the phage IME-AB2 [JX976549.1] is unclassified. An initial NCBI nucleotide BLAST analysis (BLASTN) of the complete genome sequence indicated that ZZ1 shares limited similarity with other sequenced phages. The sequences from 4 *Acinetobacter* phages, Acj9 [GenBank: NC_014663.1], Acj61 [GenBank: NC_014661.1], Ac42 [GenBank: NC_014660.1], and 133 [GenBank: NC_015250.1], were the most similar to that of ZZ1 [1]. The four *Acinetobacter* phages were recently deposited in GenBank and were previously annotated as T4-like phages [2].

The T4 phage superfamily is one of the best-characterised groups of *Escherichia coli* phages [3, 4]. Most of the known T4-like phages specifically infect certain strains of *E. coli* or other enterobacteria, but several T4-like phages can propagate in bacteria that are more phylogenetically distant, such as *Vibrio*, *Aeromonas*, *Cyanobacteria*, and *Acinetobacter* [5–9]. Although many T4-like phages have been sequenced, only a limited number of these phage genomes have been analysed in as much detail as the T4-like *Acinetobacter* phages.

Previous cross-genome comparisons of T4 and other T4-like phages [8–13] revealed that this family of phages shares a common core genome from an ancestral sequence encoding the DNA replication modules, virion structural proteins, and some conserved predicted proteins. Furthermore, some of the proteins with unknown functions are conserved in T4-like phage genomes, and some of the minor differences between these phages may be related to their adaptations to different host ranges [7, 14]. A previous study found that each of the 4 T4-like *Acinetobacter* phages, Acj9, Acj61, Ac42, and 133, has a unique set of ORFs that occupy ~35% of the genome. In other words, each represents a different type of T4-related phage genome [2]. However, to the best of our knowledge, no detailed bioinformatics analysis and comparative genomic analyses of ZZ1 compared to T4 and the other *Acinetobacter* phages have been reported. Although detailed information about the relationship between the hosts of ZZ1 and the hosts of the other 4 *Acinetobacter* phages is not available from the NCBI database and the current literature, it has been confirmed that the hosts of the 5 phages belong to *Acinetobacter* (see Table 1)*.* These phages may have evolved from a common ancestor and switched hosts during their evolution. The similarities and the differences between them can improve our understanding of their evolutionary strategy. More importantly, it would be useful to identify regions that are variable between the genomes of different T4-like phages that might underlie host-specific adaptations. Here, we describe the genome organisation of the phage ZZ1, which infects *A. baumannii* yet shares a common ancestor with the *E. coli* phage T4. The comparative genomic analyses of ZZ1, T4, and the other 4 T4-like *Acinetobacter* phages not only provides insights into viral diversity and evolution but also offers an exciting opportunity to understand the host-specific adaptation mechanism of these phages.

**Table 1:** **General genome features of ZZ1, T4 and the other completely sequenced T4-like** ***Acinetobacter*** **phages**

Phage name	Bacterial strain used in phage isolation	Accession	Size (bp)	No. of predicted CDSs	Gene density	% coding sequence	Avg gene product size (aa)	GC%
ZZ1	Acinetobacter baumannii	NC_018087.2	166687	256	1.5	93.9	203	34.41
133	Acinetobacter johnsonii	NC_015250.1	159801	257	1.6	95.5	197	39.67
Acj9	Acinetobacter johnsonii	NC_014663.1	169947	253	1.5	93.3	208	40.03
Acj61	Acinetobacter johnsonii	NC_014661.1	164093	241	1.5	92.5	209	39.01
Ac42	Acinetobacter sp.	NC_014660.1	167716	255	1.5	94.9	207	36.37
T4	Escherichia coli	NC_000866.4	168903	278	1.6	97.8	197	35.30

## Results and discussion

### Annotation of the ZZ1 genome

**Figure 1:**
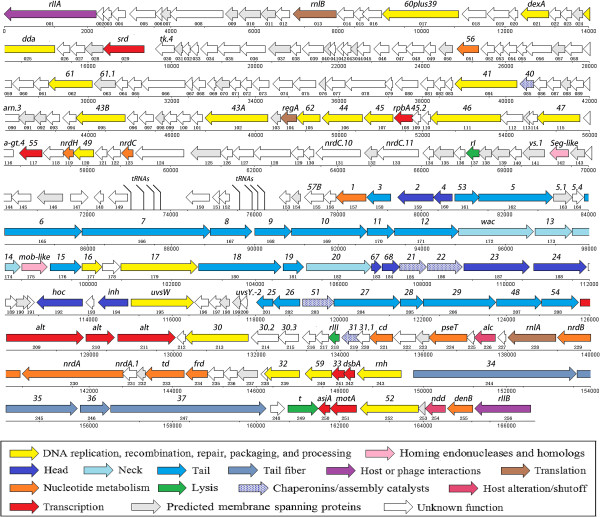
**Annotated genome map of phage ZZ1.** CDSs are labelled with the name of the closest homolog, and thus T4 nomenclature is primarily indicated (bold italics). The numbers below the CDS arrows are the CDS ids used in the text, tables, and GenBank. Following convention, the map starts at the bottom left with the *rIIA* gene and ends at the bottom right with the *rIIB* gene. The individual CDSs are depicted as arrows, with the orientation of the arrows indicating whether the genes are on the Watson or Crick strand. The colour of the arrow identifies the functional category into which the homologous T4 gene was classified [3]. The colour code for gene function is provided in the bottom of the figure.

**Figure 2:**
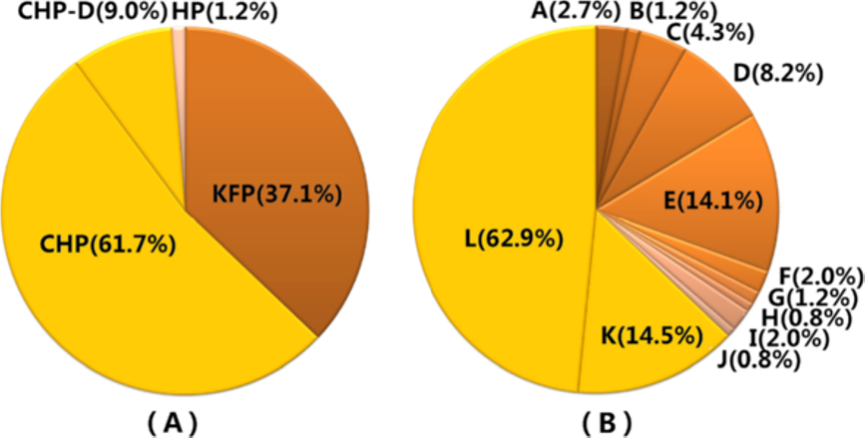
**Summary of the 256 ZZ1 genes annotated by BLASTP and Batch CD-Search analyses.** **(A)** Pie chart showing the relative abundance of genes with and without named functions, including 95 KFP (known function proteins), 158 CHP (conserved hypothetical protein), 23 CHP-D (CHP with protein domain), and 3 HP (hypothetical protein) genes. **(B)** Relative abundance of ZZ1 proteins in 12 functional categories: A, Transcription; B, Translation; C, Nucleotide metabolism; D, DNA replication, recombination, repair, packaging, and processing; E, Virion proteins; F, Chaperonins/assembly catalysts; G, Lysis; H, Host or phage interactions; I, Host alteration/shutoff; J, Homing endonucleases and homologs; K, Predicted membrane spanning proteins in unknown function proteins; L, Unknown function.

Sequence assembly yielded a closed, circular sequence with 114 bp inverted terminal repeats, indicating that ZZ1 phage particles contain linear, circularly permuted genomic DNA, similar to phage T4 particles [2]. The single copy genome is 166,687 bp. Bioinformatics methods identified 256 putative protein-coding sequences. The lengths of these CDSs range from 34 to 1,303 amino acid residues (aa) and average 203 aa. There are approximately 1.5 genes per kbp, and 93.6% of the ZZ1 genome is predicted to encode proteins (Table 1). ATG [96% (247/256)] was the predominant initiation codon. Only 4 CDSs began with GTG, and 5 began with TTG. The predominant termination codons were TAA [69% (177/256)] and TGA [27% (68/256)]. We noted that most of the significant BLASTP hits (201 CDSs, 78.5%) were proteins from phages (most of them belonging to the characterised T4-like phages), and the remaining hits were unknown proteins encoded by bacteria and other organisms. Of the 256 CDSs from ZZ1, 97 were assigned a functional annotation (see the colour code for the CDSs in Figure 1). Only 3 (1.2%) have no BLASTP matches in the non-redundant protein sequences database, indicating that genes encoding new viral proteins were revealed by the characterisation of this phage. However, further Batch CD-Search analyses suggested that 17 of the 97 genes with functional annotations lack protein domain information. Of the 256 CDSs, 156 were conserved hypothetical proteins, and only 23 of the proteins (9%) had protein domain information (see Figure 2). Further functional analysis revealed that the 97 CDSs assigned putative functions could be classified into 12 functional categories according to previous descriptions of phage T4 [3] (see Figure 1 and Additional file 1). Most of the named functional proteins are highly conserved among T4-like phages and are either structural (36 CDSs) or involved in DNA replication, recombination, repair, packaging, and processing (21 CDSs) (see Figure 2 and Additional file 1).

### Comparative genomics of T4 and ZZ1

**Figure 3:**
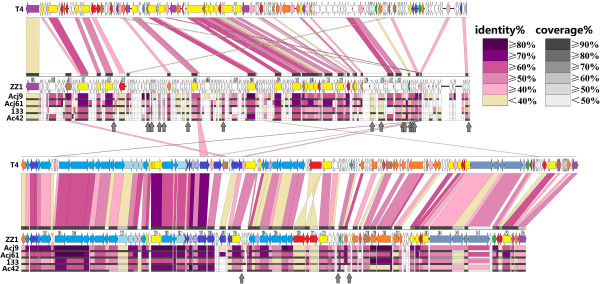
**T4-ZZ1 comparison genomic mapping and ZZ1 orthologous genes from the other 4** ***Acinetobacter*** **phages.** The genomes and coding regions of T4 and ZZ1 are drawn in parallel approximately to scale. Alignment of different genes is from T4 (upper line) and from ZZ1 (lower line). Following convention, the map starts at the top left with the *rIIA* gene and ends at the bottom right with the *rIIB* gene. Horizontal arrows indicate transcription direction. The likely function of the genes is indicated by the colour of the arrows as described in Figure 1. Genes sharing protein sequence identity are linked according to the colour key provided at the middle left. ZZ1 genes are annotated randomly with their gene order in the GenBank database. The map was artificially split into upper halves and lower halves. ZZ1 genes that are orthologous to the 4 other *Acinetobacter* phages are indicated by coloured boxes under the corresponding ZZ1 genes. The amino acid sequence identity of ZZ1 genes to these orthologous genes was also indicated by colour as described in the key provided at the upper right. The % coverage of ZZ1 genes to these orthologous genes is indicated by the grey boxes under each coloured shade or box according to the grey scale key at the upper right. The genes indicated by the grey vertical arrows share significant similarity with proteins from the 4 other *Acinetobacter* phages, but share no significant similarity with T4 proteins.

The determination of phage relatedness is not based exclusively on sequence similarity but also takes genome organisation into consideration [15]. The phage ZZ1 has similar virion morphology [1], genome size and number of CDSs compared to the coliphage T4 (168903 bp, 278 CDSs) (Figure 3). To further investigate the genomic similarities of T4 and ZZ1, we constructed comparative genome maps of ZZ1 and T4. The putative gene *rIIA* from ZZ1 was positioned on the minus strand (leftward transcription) and at the start of the map, following the T4 convention. Homologous genes are projected onto the T4 map, and the projections are colour-coded based on protein sequence similarity (see Figure 3). BLASTP analysis indicated that the ZZ1 CDS140 shows significant similarity to the putative *vs* gene (<7e-23 E value, ≥ 80% coverage, and ≥ 50% maximum identity) from the other 4 T4-like *Acinetobacter* phages, Acj9, Acj61, 133, and Ac42 (see Figure 3). However, when we compared the ZZ1 CDS140 to the set of all T4 proteins by aligning two or more sequences from BLASTP using the default parameters, no counterpart was found in the results. Further BLASTP analysis revealed that, of the 4 *Acinetobacter* phages, only phage Acj61 *vs* showed a slight similarity to the T4 *vs* (0.1 E value, 99% coverage, and 32% max identity)*.* Similar results were observed in the annotation of ZZ1 CDS156, which showed significant similarity to the putative 57A (<1e-4 E value, ≥70% coverage, and ≥37% max identity) from phages Ac42 and Acj61 although no counterpart was found in the BLASTP results when we compared ZZ1 CDS156 to the set of all T4 proteins. Thus, we annotated ZZ1 CDS140 and 156 as hypothetical proteins (see Figure 1), and of the 256 CDSs from ZZ1, only 95 (37.1%) could be assigned a functional annotation, and 158 (61.7%) were conserved hypothetical proteins (see Figure 2). Overall, such gradual evolution of the T4 genes between these closely and distantly related phages may eventually provide some interesting insights into the particular steps of genome divergence.

Notably, 110 of the 256 ZZ1 CDSs (43%) shared up to 73% amino acid sequence identity with T4 proteins (E value < 10^-6^) (see Figure 3 and Additional file 1). Long blocks of synteny containing homologous genes are interspersed with stretches that lack homology. The lower halves of the genome, which are closely related, include three separate clusters of rightward-transcribed genes, specifically: (i) a cluster of base plate wedge/head/tail genes (genes *53* to *24*), (ii) a smaller base plate hub gene cluster in its vicinity (genes *51* to *54*), and (iii) further away, a tail fibre gene cluster consisting of a few large genes (genes *34* to *t*). Two clusters of leftward-oriented non-structural genes separate the 3 rightward-oriented clusters and mainly encode proteins involved in nucleotide metabolism. The degree of sequence identity varied substantially. The degree of sequence conservation did not follow structural/non-structural gene divisions.

**Figure 4:**
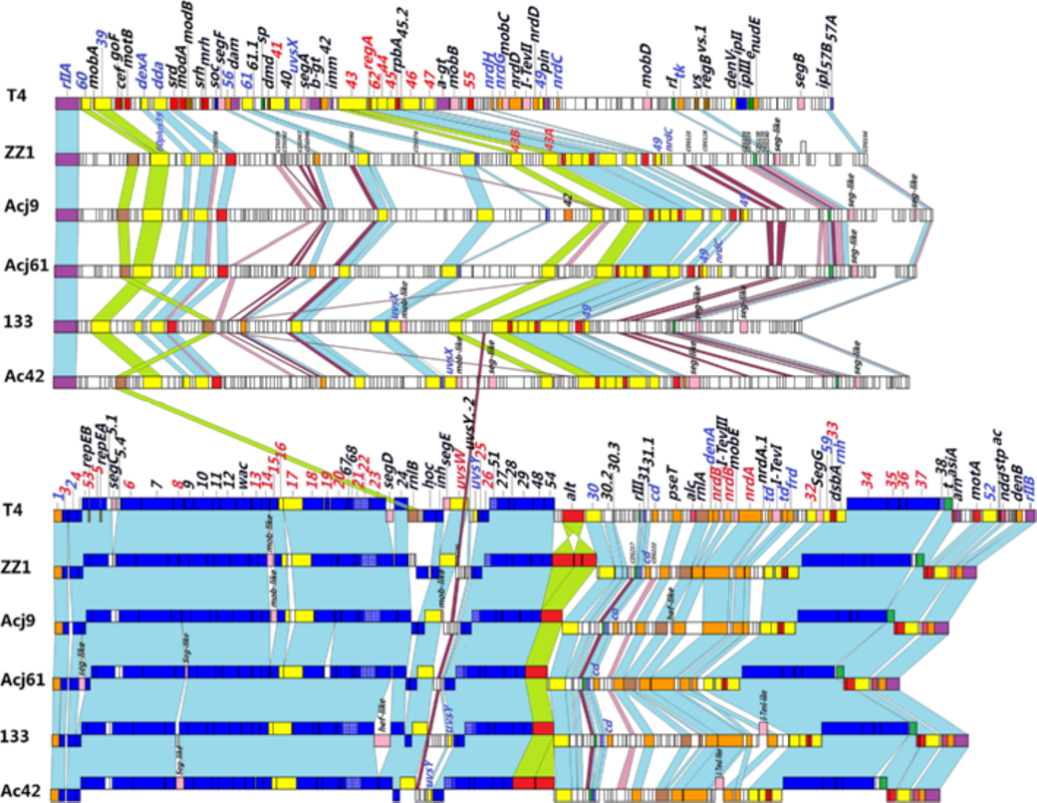
**Divergence of the core gene organisation between T4 and T4-like** ***Acinetobacter*** **phages.** The map was artificially split into upper and lower halves. The bars indicate identified ORFs and are drawn to scale. Genes transcribed in the forward direction (or transcribed rightwards) are displayed above those genes transcribed in the reverse direction (or transcribed leftwards). The functional category of T4 genes is indicated by the color-coded bars described in Figure 1. The numbers and acronyms shown directly above the color-coded bars refer to the gene names. Core and Quasicore genes described by *Petrov et al.* [2] are shown in red and blue font, respectively. Homologous signature genes, shared by T4 and the 5 T4-related *Acinetobacter* phages, are connected with light blue shading. Conversely, homologous unknown genes, which shared significant similarity with all 5 T4-related *Acinetobacter* phages but no significant similarity with T4 proteins, are linked by dark and light purple shading. Genes linked by dark purple shading are specific to the 5 T4-related *Acinetobacter* phages in the GenBank database. In addition, 4 signature T4 genes, which are linked by green shading, could reflect a dominant feature in the evolution of all 5 T4-related *Acinetobacter* phages.

The region that displays the most significant difference between ZZ1 and T4 is located in the upper halves of the genome that exclusively encode leftward-oriented, non-structural genes (with the exception of one rightward-oriented unknown gene, CDS147), including a large DNA replication module. Large regions of nonaligned genome segments were thought to have resulted from genetic gains, losses, or replacements, i.e., events similar to those that have shaped the evolution of all microbial genomes in nature. For example, ZZ1 *rIIA.1.60, 60.1* and *mobA* are flanked by 14 unknown genes and 19 genes were deleted between ZZ1 *vs.1* (CDS141) and *segB* (CDS142). Further alignment and comparison of T4 genes that are homologous to ZZ1 revealed that the order and transcriptional direction of T4 genes are broadly maintained in the ZZ1 genome, which helped provide the phylogenetic distance between ZZ1 and T4. There were three major differences: 1) the *rnlB* gene was at the junction of *24* in the lower genome half in T4 but was adjacent to the CDS012 in the upper genome half of ZZ1 (see Figure 3); 2) the topoisomerase gene (*39 + 60*) and the DNA polymerase gene (*43*) were split and intact, respectively, in T4 but intact and split, respectively, in phage ZZ1; and 3) a cluster of *Alt* genes including three putative *Alt-like* genes differs between T4 and ZZ1. As shown in Figure 3, the transcriptional direction of the three ZZ1 *Alt-like* genes was different from the T4 *Alt,* revealing an unusual genomic organisation for the *Acinetobacter* phage ZZ1. Moreover, the first *Alt* counterpart (CDS209) is 695 aa in length and shares 98% coverage and 28% amino acid identity with the T4 *Alt* (total 682 aa in length). The second *Alt-like* gene (CDS210) is 229 aa in length and shares 35% amino acid identity with the T4 *Alt* segment that extends from 1 to 217 amino acids, and the third *Alt* analogue (CDS211) is 463 aa in length and shares 33% amino acid identity with the T4 *Alt* segment that extends from 409 to 643 aa*.* Apparently, the last two ZZ1 *Alt-like* genes resulted from an even splitting event from the ancestral *Alt* gene (see Figure 3). The duplication of the *Alt* gene was also present in the genomes of the *Acinetobacter* phage Ac42 (see Figure 4), the coliphage JS98 (NC_010105.1), and the *Enterobacteria* phage Bp7 (NC_019500.1), but the splitting of the *Alt* gene was not observed in other T4-like phages.

T4-like phages were often shaped by gene duplications. For example, in T4, genes *23* and *24* share 29% amino acid identity [3], and 16 proteins (mostly hypothetical) were identified as likely to have been duplicated in the KVP40 lineage [8]. To further examine duplications, we aligned genes from ZZ1 with other ZZ1 genes using BLASTP at the amino acid level and BLASTN at the DNA level. Those with better matches (based on *E* value) were considered candidates for lineage-specific duplications. Using this method, 14 proteins were identified as likely to have been duplicated in the ZZ1 lineage (data not shown). We further reasoned that if the homologous genes are the result of duplication of an ancestral gene, and if the different degrees of sequence diversification of these duplicated genes resulted from the accumulation of nucleotide point mutations resulting from differential selection pressure, then the genes with lineage-specific duplications should have significant nucleotide sequence identities. However, although the duplicated genes have significant amino acid identities, no significant nucleotide sequence identities were observed. Thus, the similarity of the genes is more likely to be the result of convergent evolution than the result of duplication of an ancestral gene. For example, ZZ1 *23* and *24* share 25% amino acid identity but negligible nucleotide sequence identity (data not shown). The *24* protein plays an interesting role in T4 head maturation. The T4 prohead consists of an outer shell (*23*) and an inner scaffold (*22*). When *24* is added to the prohead, maturation cleavage occurs: *23* loses its N-terminal portion, whereas *22* is degraded completely [16]. In addition, in phage KVP40, the duplication of three genes encoding proteins associated with the phage tail or the tail fibre (*gp12*, *gp19*, and *gp37*) suggests added flexibility in the range of host adaptation and the infection process [8]. In our study, duplication was observed in three putatively duplicated *gp34* (CDS244) homologues (*gp37*, *gp12*, and *gp36* compared to CDS247, CDS171, and CDS246, respectively), which encode tail fibre-associated proteins and share 29-30% amino acid identity with ZZ1 *gp34*. Overall, splitting and/or duplication followed by sequence diversification and new gene insertion might be a mode of T4-like phage evolution.

### Similarities and distinctions in the genome features of the *E. coli*phage T4 and the T4-like *Acinetobacter*phages

**Figure 5:**
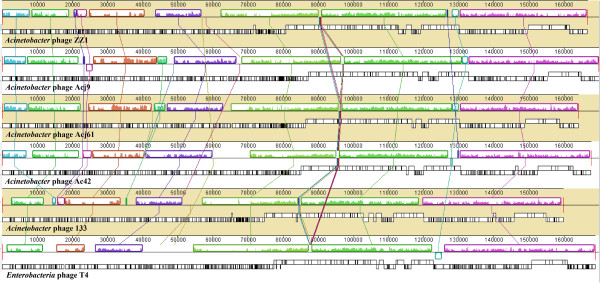
**Multiple genome alignment and the genomes of ZZ1 and 5 T4-like phages.** Six genomes were compared using Mauve software, and similarity profiles were generated. Boxes with identical colours represent local colinear blocks (LCB) indicating homologous DNA regions shared by two or more genomes without sequence rearrangements. The relative locations of orthologous genes, shown by open boxes, reveal a high degree of synteny. White regions within LCBs represent nonidentical sequences.

Alignment of the ZZ1 genome with the T4-like phages in the Tulane database showed that ZZ1 was closely related to 4 *Acinetobacter* phages. Based on the total search score, the closest was Acj9, followed by Acj61, Ac42, and 133. No other *Acinetobacter* phages were uncovered. In DNA sequence dot plots, we observed a frequently interrupted but straight diagonal line between ZZ1 and the 4 phages (data not shown). Overall, their genomes are colinear but are frequently interrupted by replacements with unrelated genome segments of comparable lengths, especially in the first halves of their genome. Further comparison of these genome sequences (including T4) at the DNA level using Mauve showed remarkable synteny in five large conserved regions (>10,000 bp) (see Figure 5). The most conserved syntenic region covered the morphogenesis module as well as the DNA replication and metabolism module, which are consistent with previous analyses [2, 14].

Protein-by-protein comparison of the 5 phages using BLASTP and CoreGenes revealed that ZZ1 shares 179 (69.9%) protein homologues with *Acinetobacter* phage Acj9, 164 (64.1%) with Acj61, 157 (61.3%) with 133, and 143 (55.9%) with Ac42, although ZZ1 shares only 110 protein homologues with coliphage T4. In contrast, 101 protein homologues are present in T4 and in all 5 *Acinetobacter* phages, whereas 119 homologous genes are present in all 5 of the *Acinetobacter* phages (46.3 to 49.3% of their protein-coding genes), which is further evidence that ZZ1 and the 4 other *Acinetobacter* phages belong to the same genus (see Figure 3).

The full portrait of the T4 phage superfamily that has emerged from the many reported genomic comparisons [2, 9–11] is that the T4 superfamily can be distilled down to core signature genes, which are known as the “Core Genome” of the T4-related phages or T4-like Viruses [2]. The Core Genome primarily includes homologues of essential T4 genes, such as the virion structure and DNA replication genes. In contrast, the hyper plastic regions (HPRs) contain mostly novel genes of unknown function and origin [7, 14]. The Core Genome of the T4-related phages has been considered to consist of two genetic components: one highly resistant component was termed the Core genes, which are essential for all known conditions; and the other component, which is somewhat permissive to attrition in evolution, was termed the Quasicore genes and can be substituted or circumvented in certain genetic backgrounds of phage and/or bacterial hosts [2]. The genetic background for the Core Genome can vary considerably between T4 relatives, and thus, the number of the core genes that constitute the Core Genome depends on the precise subset of phages considered, which constitutes an important criterion for distinguishing between close and distant relatives [14]. A closer look at the components and organization of the Core Genome in the 6 T4-like phages (see Figures 3 and 4) shows that all 5 *Acinetobacter* phages share a Core Genome with T4 interrupted by several HPRs, which are where most of their divergence occurs. This finding is consistent with the previous observations of T4-like phages [2, 7, 11, 13, 14, 17]. All of the Core genes described by Petrov *et al.* exist in all 5 T4-related *Acinetobacter* phages and are indicated by the red font directly above the coloured genes bars in Figure 4. The topology of the set of T4 phage Core genes is also shared by all 5 T4-related *Acinetobacter* phages. Moreover, we observed that almost all the Quasicore genes described by Petrov *et al.* ( by the blue font directly above coloured genes bars in Figure 4) were shared by all 5 T4-related *Acinetobacter* phages, except for *uvsX* (RecA-like recombination protein)*, uvsY* (*uvsX* helper protein)*, nrdC* (thioredoxin)*, nrdG* (subunits of an anaerobic ribonucleotide reductase complex)*, tk* (thymidine kinase)*, 49* (Endonuclease VII, required for recombination and DNA packaging), *cd* (dCMP deaminase)*,* and *denA* (Endonuclease II). These genes are permissive to attrition in evolution of all 5 T4-related *Acinetobacter* phages (see Figure 4). In addition, nearly half of the other T4 signature genes (indicated by the black font directly above coloured genes bars in Figure 4) are shared by all 5 T4-related *Acinetobacter* phages, including the gene topology. Thus, all 5 T4-related *Acinetobacter* phages use similar mechanisms to control propagation in their hosts. These phages all shared a common ancestral genome but, during the course of evolution, they have modified it in numerous ways. These conserved T4 genes allowed the 5 T4-related *Acinetobacter* phages to conserve their highly successful virion design and mode of replication. Notably, several major distinctive genome features in all 5 T4-related *Acinetobacter* phages (indicated by the green shading in Figure 4) could reflect a dominant feature in the evolution of all 5 T4-related *Acinetobacter* phages.

The most significant difference between the 5 T4-related *Acinetobacter* phages and T4 is in a series of HPRs, which are interspersed between the conserved T4 core sequences and especially enriched in the upper genome halves of all 5 T4-related *Acinetobacter* phages. These regions vary greatly in gene number and content. The HPRs were primarily composed of genes of unknown origin, but they do contain some identifiable sequences from bacteria and unrelated phages. Thus, the HPRs have been predicted to be the result of an evolutionary history of isolation within distinct hosts and extensive lateral gene transfer (LGT), i.e., importing genes or exchanges with diverse biological entities in nature [7, 18]. Our observations showed that all of the genes lack T4 homologues in the 5 T4-related *Acinetobacter* phage genomes and cannot be assigned a putative function, most of which likely appear to be homologous genes from other phages, bacteria or other organisms. Notably, of the 119 ZZ1 genes that have significant homology with genes present in 4 of the *Acinetobacter* phages, 31 have unknown functions. Of these 31 genes, 18, which are indicated by the vertical grey arrows in Figure 3 (the counterparts of the 18 ZZ1 genes in the 4 other T4-related *Acinetobacter* phages are linked by dark or light purple shading in Figure 4), share no significant similarity to T4 proteins. Moreover, most of these homologous genes maintain a consistent order and content in the 5 T4-related *Acinetobacter* phages (see Figure 4). Further, BLASTP analyses suggested that 9 of the 18 ZZ1 genes (CDS042, CDS047, CDS049, CDS060, CDS125, CDS128, CDS138, CDS196, and CDS217), whose counterparts are linked with dark purple shading in Figure 4, lack significant matches to any of the phage sequences in GenBank except for the 4 other *Acinetobacter* phages (E value < 10^-5^) and a few other organisms (E value > 0.1). In particular, 7 (CDS042, CDS047, CDS049, CDS060, CDS138, CDS196, and CDS217) share high similarities (>50%) with the other 4 *Acinetobacter* phages.

The diversity among phage “Pangenome” (the union of all the different naturally occurring ORFs that exist in T4-related phages [2]) is a reflection of the adaptations of a phage ancestor to a variety of evolutionary challenges, including encountering new host environments. LGT most certainly has played a role in such adaptations, and these adaptations may have been facilitated in part by certain particularities of the T4 phage recombination and gene expression systems [7, 14]. However, to date, there are few clues about the agents that might mediate such a transfer. T4 recombines most efficiently early in the infection before the host genome is degraded, and this process could facilitate the acquisition of host genes by the phage [19]. As little as 50 bp of homology (perhaps less) is sufficient for T4 recombination system to recombine at reasonable frequencies [20]. Thus, “semilegitimate” recombination that relies on the small but generally conserved regulatory signals in the intergenic regions (promoters, translation initiation regions, transcription terminators, etc.) could perhaps have mediated the acquisition of such foreign genes [21]. Once the foreign genes are acquired, the T4 expression system could exploit their endogenous bacterial promoters because these sequences are very similar to the early promoter sequences of the T4-type phages [11, 22]. Overall, different phages sharing a common ancestor undergo a specific and similar adaptation process for different bacterial strains that belong to the same genera or species. The existence of these highly conserved unknown homologous genes specific to *Acinetobacter* phages in HPRs could reflect the complex interactions of *Acinetobacter* phages with conserved cell components that are specific to *Acinetobacter* bacterial hosts and are distantly related to *E. coli* or other bacterial hosts. Theoretically, the uniqueness of homologous genes or certain sequences in specific phage genomes or lineages might help distinguish between the different clusters or types of phage and help predict the bacterial host range when treating the corresponding bacterial genera clinical isolates. Additional research is required to elucidate the highly conserved genes that are specific to *Acinetobacter* phages.

### GC skews in the ZZ1 genome

Phage ZZ1 DNA contains only 34.4% GC, which is slightly lower than the value (38.9% to 39.2%) observed in sequenced *A. baumannii* strains [23–26]. To date, it is the lowest reported GC content for sequenced T4-like *Acinetobacter* phages (see Table 1). The GC content of ZZ1 is comparable with that of the *Enterobacteria* phage T4 (35.3%). The mol% GC of the T4 genome is also substantially lower than that of its host (approximately 50% GC) [3]. The difference is in disagreement with previous studies suggesting that the GC content of phage genomes such as the mycobacteriophages [27] and *Staphylococcus aureus* phages [28] have a GC content similar to their respective hosts.

Although ZZ1 has a lower GC content, 12 of the predicted phage ZZ1 genes contain more than 40% GC, including one major head gene (*23* counterpart/CDS187), three tail fibre genes (*12* counterpart/CDS171*, 37* counterpart/CDS247, and *36* counterpart/CDS246), one nucleotide metabolism gene (*cd* counterpart*/*CDS221), and seven unknown genes. Notably, the GC content of the ZZ1 *23* gene is 40.5%, which is significant lower than the local GC content of T4 *23* (45%) and the T4-like phage JS98 *23* (47.6%) [17]. Gene *23* has the highest proportion of codons that are translationally optimal for the host (65%) in keeping with its very high level of expression; approximately 1,000 copies of the protein are needed per synthesised phage particle [3]. Interestingly, the GC content of the ZZ1 host, *A. baumannii* strains, is approximately 39% and is lower than the GC content of the host strains for T4 and JS98, which is 50.8%. If the GC content of phages and their hosts become more similar during extended co-evolution, the difference between the GC content of ZZ1 gene *23* and the GC content of gene *23* in the phage T4 and JS98 presumably reflects the differences in host range, GC content, and life history of the respective hosts. Thus, the low GC content of ZZ1 *23* suggests that phage ZZ1 might have acquired the ability to infect *A. baumannii* strains over a long period of time*.*

**Figure 6:**
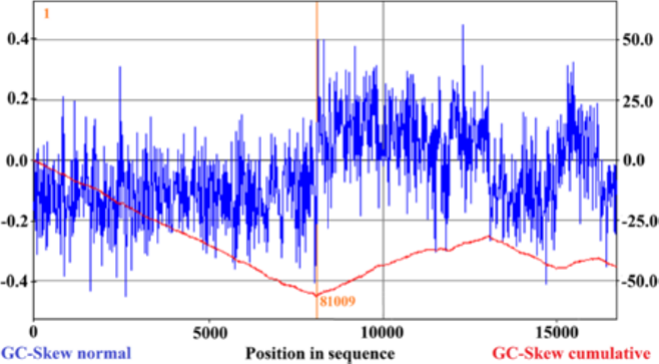
**Cumulative GC skew analysis of the genome sequence.** The global minimum and maximum are displayed in the cumulative graph. The minimum and maximum of a GC-skew can be used to predict the origin of replication (nt 81009) and the terminus location (nt 1).

One region was found to be devoid of CDSs, extending from 72,968 to 76,602. In an effort to determine whether this region served as the replication origin, we used GC-skew analysis [29]. A putative origin of replication in the region of nt 81009 and a putative terminus of replication in the region of nt 1 were revealed (see Figure 6). The putative origin of replication is close to but not within the CDS-devoid region. However, tRNA detection showed that the tRNA genes from phage ZZ1 were encoded between CDS149 and 153, and 2 clusters of tRNAs (4 tRNAs in each) were located near the three adjacent genes, CDS150, 151, and 152. The two regions were in this CDS-devoid region (as shown in Figure 1). A previous study revealed that the phage ZZ1 had a very short latent period of 9 min [1]. The large number of tRNA genes and their strategic locations might enable the phage to translate its sequence more efficiently, reducing its latency time and increasing its reproduction rate and thus its infectivity.

### Codon usage and tRNAs

**Figure 7:**
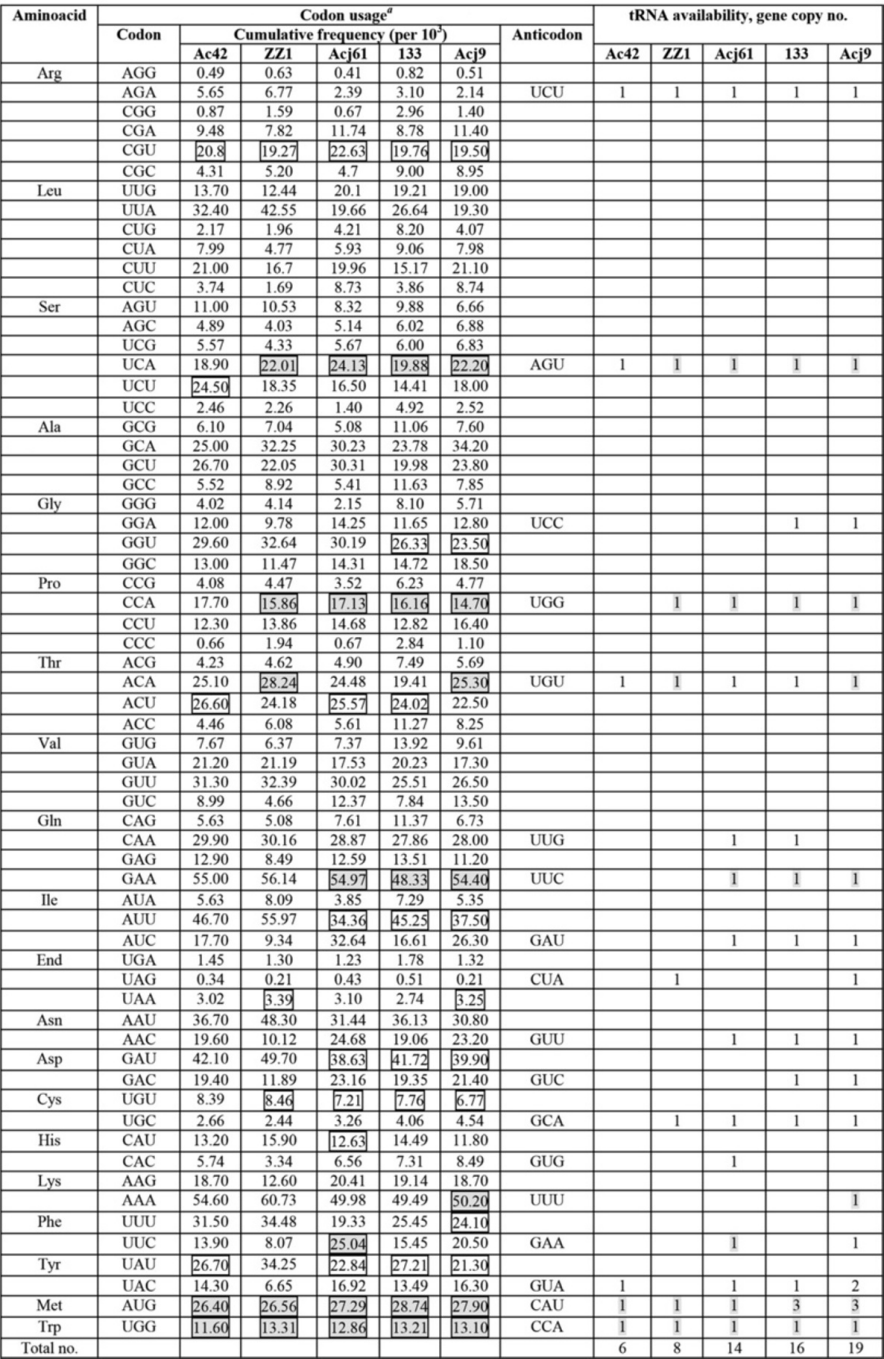
**Codon usage and tRNA availability in** ***Acinetobacter*** **phages.** The total number of codons is 52,152 for phage ZZ1 (256 genes), 52,870 for phage Acj9 (253 genes), 50,605 for phage Acj61 (241 genes), 52,929 for phage Ac42 (255 genes), and 51,005 for phage 133 (257 genes). Optimal codons that have relative phage-encoded tRNA are indicated with black boxes. Phage-encoded tRNAs related to the highly used codons are shaded grey, as is the corresponding codon.

Eight tRNA genes were predicted in the ZZ1 genome (see Table 2). The T4 genome also has 8 tRNA genes, whereas Ac42 has slightly fewer (6), and others have substantially more including phage 133 (16), Acj9 (19), and Acj61 (14), as shown in Figure 7. The different numbers and types of tRNAs present in the 5 *Acinetobacter* phages are consistent with a previous study of tRNAs in T4-like phages [7, 13]. In addition, although ZZ1 has a low mol% GC content (34.4%), 4 (50%) out of the 8 tRNAs recognise codons with A in the third position (see Figure 7).

**Table 2:** **tRNA genes from ZZ1 and homologous tRNAs from 4 other** ***Acinetobacter*** **phages and T4**

Locus tag	tRNA type (anticodon) ^a^	Location (bp)	Length (bp)	G + C content	Similar hit in 4 ***Acinetobacter***phages and T4 ^b^			
					Phagename	tRNA type (Anticodon)	tRNA location	(Coverage/identities)
ZZ1t001	Trp (CCA)	73037-73108	72	55.60%	Acj9	Cys (GCA)	81632-81707	51%/80%
					Acj9	Trp (CCA)	78801–78872	100%/89%
					133	Trp (CCA)	66679–66754	100%/90%
					Acj61	Cys (GCA)	80595-80670	68%/79%
ZZ1t002	Pro (TGG)	73349-73425	77	50.60%	Acj61	Pro (TGG)	79527-79603	93%/75%
					133	Pro (TGG)	67417-67493	64%/76%
ZZ1t003	Ser (TGA)	73639-73733	95	61.10%				
ZZ1t004	Thr (TGT)	73887-73962	76	53.90%	Acj9	Thr (TGT)	79955-80030	100%/80%
					Acj9	Lys (TTT)	79870–79945	72%/75%
					Ac42	Thr (TGT)	79430–79505	53%/83%
					133	Ile (GAT)	67500–67575	72%/75%
					T4	Pro (TGG)	72206-72280	77%/83%
ZZ1t005	Cys (GCA)	75677-75753	77	57.10%	Acj61	Cys (GCA)	80595-80670	79%/89%
					133	Cys (GCA)	69475–69550	79%/85%
					Acj9	Cys| (GCA)	81632-81707	72%/86%
ZZ1t006	Met (CAT)	75945-76021	77	54.50%	Acj9	Met (CAT)	81715-81790	100%/84%
					133	Met (CAT)	69740–69815	100%/84%
					133	Met (CAT)	66679-66754	55%/83%
ZZ1t007	Arg (TCT)	76027-76102	76	53.90%	133	Arg (TCT)	68572-68647	100%/88%
					Acj9	Arg (TCT)	79288–79363	92%/81%
					Acj61	Arg (TCT)	80679-80755	71%/:87%
ZZ1t008	Sup (CTA)	76196-76269	74	51.40%	Acj9	Pyl (CTA)	82721-82793	98%/75%

^a^Two tRNA scan tools, tRNAscan-SE and ARAGORN, were used for prediction. All of the predicted tRNA genes are located on the minus strand of the ZZ1 genome.

^b^tRNAs homologous to ZZ1 in other phages revealed by nucleotide comparison (using BLASTn with the following cut-offs: coverage, >50% and E value, ≤10^-6^) are also located on the minus strand of the phage genomes.

The exact function of phage-encoded tRNAs in the phage-infected cell is still not clear. They might be involved in the adaptation of the host translation apparatus to the demands of the phage codon usage pattern. In other words, the tRNAs overcome the phage codon usage problem, and the presence of tRNAs in a phage genome has been suggested to compensate for differences in codon usage between the phage and the host, corresponding to codons that are expected to be poorly translated by the host machinery [30]. Previous studies indicated that T4-encoded tRNAs are related to codons that are highly used in T4 genes but rarely used in the host and that the phage tRNAs can enhance the low expression of T4 late-stage protein genes through optimal codon usage in translation [3, 31]. Similarly, analysis of the ZZ1-encoded tRNAs indicated that over half of them (5 out of 8) might be related to the optimal codon usage of ZZ1 proteins (Figure 7). However, the codon usage frequencies of the 4 other *Acinetobacter* phages suggested that phage-encoded tRNAs might be unrelated to decoding the relatively more frequent codons in all of the genes (Figure 7). Thus, the functional role of the tRNA genes for these phages remains unclear. Although the types and numbers of phage-encoded tRNAs differ between the 5 phages, some consistency was observed. For example, all 5 phages encode Met-tRNA and Trp-tRNA, which are related to optimal codon usage, but Arg-tRNA, which was not related to optimal codon usage, was also common to all 5 phages (see Figure 7). Of the 8 tRNA genes in ZZ1, 6 (75%) shared up to 89 and 90% nucleotide sequence similarity to tRNAs from phage Acj9 and phage 133, respectively; 4 (50%) shared up to 89% nucleotide sequence similarity to the tRNAs from phage Acj61; and only 1 tRNA showed 83% sequence similarity with the tRNA-Thr from phage Ac42 (see Table 2). In addition, the ZZ1-encoded tRNA-Thr gene shared 83% identity with the T4-encoded tRNA-Pro gene (see Table 2). The high degree of conservation of some of the tRNAs suggests an important functional role. The number and type of homologous tRNA appears to confirm the relationship between ZZ1 and the other phages, suggesting some elements of vertical evolution in these tRNAs.

### Mobile elements

The homing endonuclease genes are not genuine phage DNA; rather, they belong to intron-associated selfish DNA elements [32, 33]. There are 7 *seg*, 5 *mob*, and 3 *intron nuclease* (*I-TevI* to *-III*) genes in the T4 genome [3], which have efficiently invaded the T4 genome and are also frequently found in the DNA transaction module opposite the tail fibre cluster (*I-TevI* and *segG*) and the DNA replication module (*segA*, *mobB*, *mobC*, and *I-TevII*) (Figure 3). Inconsistent distribution of these elements has been described for T2, T4 and T6 phages [34]. One notable difference between the T4 and ZZ1 genomes is that ZZ1 appears to lack counterparts in the corresponding regions, except for two mobile genes, a *seg-like* gene and a *mob-like* gene. The ZZ1 *seg-like* mobile endonuclease (CDS142), which is located in the DNA replication module, has 34 to 54% similarity to 6 *seg* genes in T4 (*segA, segB, segF, segC, segD,* and *segE*). However, the ZZ1 *seg-like* gene has higher similarity with the *seg* counterparts of the other 4 *Acinetobacter* phages: 73% amino acid identity with the *seg* counterpart from Acj9, and 56 to 58% identity with *seg* counterparts from Acj61, 133 and Ac42. The ZZ1 *mob-like* homing endonuclease (CDS175), which was found in the cluster of neck genes, lacks sequence similarity with the corresponding T4 genes. Nevertheless it has 52% sequence similarity with the mob counterparts from Acj9 and 33 to 39% sequence similarity with the *mob* counterparts from 133 and Ac42. The other 4 *Acinetobacter* phages also lack mobile elements in the corresponding locations. Although each of them has two to four *seg* genes, no *mob-like* genes were found in phage Acj61, only one *mob-like* gene was found in phage 133 and Ac42, and only two *mob-like* genes were found in Acj9 (see Figure 4). T4 has three self-splicing group I introns, one each in *td*, *nrdB*, and *nrdD* [3] (see Figure 4). A few group *I-like* introns have been found in other phages, such as T4, the *S. aureus* phage Twort [35], the *Bacillus* phages I-BasI and I-HmuI [36], the *Lactobacillus* phage LL-H, and the *Lactococcus* phage r1t [37]. However, none of the three T4 *intron nuclease* genes were found in phage ZZ1, Acj61, or Acj9, and only one *I-TevI-like* homing endonuclease from the GIY-YIG family was found in the 133 and Ac42 genome (see Figure 4). These mobile element genes do not commonly exist in the T4-like phages, and the differences in mobile elements between the 5 *Acinetobacter* phages and T4 phage genomes are consistent with the results for another T4-like phage [7, 13] Apparently, there are ill-defined barriers to intron promiscuity in bacteriophages [38, 39].

## Conclusions

We determined and revised the complete genome sequence of the phage ZZ1 that infects pathogenic *A. baumannii* strains*.* A total of 256 potential proteins and 8 tRNAs were predicted. The BLASTP analysis reveals that only a small portion of its proteins (110 proteins, 43%) are clearly related to coliphage T4 proteins and share up to 73% protein sequence identity with the corresponding T4 proteins. Further analysis revealed that 179, 164, 157, and 143 proteins from ZZ1 share up to 86, 85, 81, and 83% amino acid identity, respectively, with *Acinetobacter* phage Acj9, Acj61, 133, and Ac42 proteins, respectively. Nine ZZ1 genes lack significant matches to any of the phage sequences in the GenBank database except for the 4 other *Acinetobacter* phages. In addition, the high degree of conservation of some tRNAs between ZZ1 and the other T4-like phages suggests that they may have an important functional role in addition to overcoming the phage codon usage problem. The number and identity of the homologous tRNAs supports the evolutionary relationship between ZZ1 and the other phages, suggesting some elements of vertical evolution in these tRNAs. Overall, although more than 200 T4-like phages have been examined [4, 13], only a very limited number of the T4-like *Acinetobacter* phage genomes have been explored or exploited. As additional genome sequencing of *Acinetobacter* phage species are completed, more *Acinetobacter* phages similar to T4 might be discovered. Host-specific adaptation mechanisms might be revealed by a more comprehensive understanding of the genomic diversity within the *Acinetobacter* bacteriophage population in the future*.*

## Methods

### Bacterial and phage strains

*A. baumannii* AB09V was isolated from the sputum of one hospitalised patient at the Henan Province People’s Hospital in Zhengzhou, China. After obtaining the approval of the Life Science Ethics Committee of Zhengzhou University and written informed consent, sputum samples were collected for the purposes of this study. The automated system BD Phoenix (Becton Dickinson Diagnostic Systems, Sparks, MD, USA) was used on clinical samples for the identification of bacteria and for antibiotic susceptibility tests. Moreover, as the host of phage ZZ1, AB09V has been further confirmed as *A. baumannii* using sequence information derived from the 16S rRNA gene in our previous work [1]. Phage ZZ1 was propagated in the *A. baumannii* strain AB09V as previously described [1].

### Phage genome resequencing

The nucleic acid from the phage ZZ1 was extracted and purified from phage lysate using a MiniBEST Viral RNA/DNA Extraction Kit Ver. 4.0 (TAKARA BIO Inc., Tokyo, Japan) according to the manufacturer’s protocol. Phage DNA was then sent to Zhejiang California International NanoSystems Institute for sequencing. Phage DNA was fragmented with a Covaris S220. After end-repair and ligation of adaptors, libraries were amplified by polymerase chain reaction (PCR), purified with a QIAquick® PCR extraction kit (Qiagen, Venlo, The Netherlands), and sequenced on an Illumina Solexa Sequencing platform (Illumina, San Diego, USA) with a read length of 2 × 250. The whole genome sequences of ZZ1, with a total length of 166,682 bp, were obtained [1]. However, when we analysed in further detail the entire ZZ1 genome to understand the genetic characteristics of this phage, we found many split genes, which were considered to be due to errors from the sequence read assembly. Thus, in this study, we amended these errors by PCR + sequencing directly from phage genomic DNA. Ultimately, the length of revised single copy ZZ1 genome was 166,687 bp.

### Bioinformatics analyses

Open reading frames (ORFs) were identified using two bioinformatics software programs, GenMarkS [40] and fgenesV0 (http://linux1.softberry.com/berry.phtml). The predicted translational regions were corroborated by manual inspection. These CDSs were considered valid if they possessed at least 30 amino acids (aa), showed a putative ribosome binding site (RBS) at a convenient distance, and began with an AUG, UUG, or GUG codon. The BLASTP program (version 2.2.15) [41] from NCBI was used to search for sequence similarity of the predicted CDSs against the NCBI nonredundant protein sequence database. Protein domain searches were conducted through BLAST and by using Batch CD-Search [42]. Cumulative GC skew was measured with GenSkew at http://genskew.csb.univie.ac.at/. ZZ1 phage codon usage was analysed with the CUSP and CAI programs of the EMBOSS package, version 6.2.0 [43]. tRNAs were identified using the tRNAscan-SE server [44] and confirmed using the ARAGORN program [45] as well as by nucleotide comparison using BLASTN (http://blast.ncbi.nlm.nih.gov/Blast.cgi). TMpred was used to identify membrane-spanning regions in CDSs (http://www.ch.embnet.org/software/TMPRED_form.html).

### Comparative genomics

The phage genome sequences downloaded from NCBI and the Tulane T4-like genome (http://phage.bioc.tulane.edu) are listed in Table 1. Basic genome features of all of the phages, such as their host specificity, taxonomy, and genome molecular type, were also collected from the NCBI database and the literature. The dot matrix view from NCBI was used to examine whole-genome similarity using the default parameters. Further genomic comparisons at the nucleotide level were made with Mauve 2.2.0 [46] using a progressive alignment with default settings. Comparisons at the proteomic level were made using CoreGenes (http://binf.gmu.edu:8080/CoreGenes2.0/custdata.html) [47].

### Availability of supporting data

The ZZ1 sequence data were deposited at GenBank under accession number NC_018087.3, which replaces the previous accession number. The CDS prefix indicates a predicted protein coding sequence, followed by numbers specifying the locus tag in the GenBank file (i.e., CDS001 is locus ZZ1p0001). The GenBank accession numbers of the other phage genomes analysed in this study are listed in Table 1.

## Electronic supplementary material

Additional file 1: **CDSs of the ZZ1 genome and best BLASTP hits.** (XLS 165 KB)

## Abbreviations

CDS: Coding sequence
HPR: Hyper plastic region
LGT: Lateral gene transfer
ORF: Open reading frame
RBS: Ribosome binding site.
